# Sodium fluorocitrate having protective effect on palmitate-induced beta cell death improves hyperglycemia in diabetic db/db mice

**DOI:** 10.1038/s41598-017-13365-5

**Published:** 2017-10-10

**Authors:** Ik-Rak Jung, Sung-E. Choi, Seung A. Hong, Yoonjung Hwang, Yup Kang

**Affiliations:** 1 Department of Physiology, Ajou University School of Medicine, Suwon, Gyunggi-do 443-749 Republic of Korea; 2 Department of Biomedical Science, The Graduate School Ajou University, Suwon, Gyunggi-do 443-749 Republic of Korea

## Abstract

Beta cell loss and insulin resistance play roles in the pathogenesis of type 2 diabetes. Elevated levels of free fatty acids in plasma might contribute to the loss of beta cells. The objective of this study was to find a chemical that could protect against palmitate-induced beta cell death and investigate whether such chemical could improve hyperglycemia in mouse model of type 2 diabetes. Sodium fluorocitrate (SFC), an aconitase inhibitor, was found to be strongly and specifically protective against palmitate-induced INS-1 beta cell death. However, the protective effect of SFC on palmitate-induced cell death was not likely to be due to its inhibitory activity for aconitase since inhibition or knockdown of aconitase failed to protect against palmitate-induced cell death. Since SFC inhibited the uptake of palmitate into INS-1 cells, reduced metabolism of fatty acids was thought to be involved in SFC’s protective effect. Ten weeks of treatment with SFC in db/db diabetic mice reduced glucose level but remarkably increased insulin level in the plasma. SFC improved impairment of glucose-stimulated insulin release and also reduced the loss of beta cells in db/db mice. Conclusively, SFC possessed protective effect against palmitate-induced lipotoxicity and improved hyperglycemia in mouse model of type 2 diabetes.

## Introduction

Type 2 diabetes (T2D) is developed when pancreatic beta cells fail to secrete sufficient amounts of insulin to meet the metabolic demand due to insulin resistance^1^. Insulin insufficiency is thought to be caused by reduction in the mass of beta cells and secretory function. Histological studies have confirmed the loss of beta cell mass in patients with T2D^2,3^. In particular, obesity-induced insulin resistance increases the level of free fatty acid in the plasma. It may induce beta cell failure through its toxicity to beta cells, thereby aggravating glycemic control^4,5^.

It is known that saturated fatty acids such as palmitate and stearate can induce apoptotic death in beta cells *in vitro* (lipotoxicity)^6,7^. Several intracellular mediators involved in fatty acid-induced lipotoxicity have been reported. For example, nitric oxide and reactive oxygen species as activators of oxidative stress signals have been suggested as mediators of fatty acid-induced beta cell death^6,8,9^. Insufficient activation of autophagy has been found to be involved in fatty acid-induced lipotoxicity^10^. Increased intracellular calcium through excessive cellular calcium influx and endoplasmic reticulum (ER) calcium efflux and subsequent activation of apoptotic calcium signals is also involved in lipotoxicity^11,12^. In particular, prolonged activation of unfolded protein response in ER has been reported to be a critical mediator in fatty acid-induced lipotoxicity^13–15^.

Although the reason why various stress signals involved in apoptotic death are activated in fatty acid-exposed beta cells has not been clearly determined, derangement of fatty acid metabolism in cells appears to be involved in the initiation of stress signals. Inhibition of acyl-CoA synthetase as the first step of fatty acid metabolism has been found to be protective against palmitate-induced lipotoxicity^6^. Lipid derivatives such as diacylglycerol, lysophosphatidic acids, and ceramide synthesized through augmented lipogenesis have been initially reported to play a role in fatty acid-induced lipotoxicity since increased fatty acid oxidation through treatment with AMP-activated kinase (AMPK) activator and peroxisome proliferator-activated receptor (PPAR) alpha agonist could prevent lipotoxicity^5,16^. On the other hand, it has been reported that augmentation of lipogenesis can protect against palmitate-induced lipotoxicity if lipogenesis is stimulated in conjunction with stimulation of oxidation metabolism^17^. In particular, Prentki *et al*. have suggested that deregulated glycerolipid/fatty acid cycling is involved in lipotoxicity^18^. Mitochondrial dysfunction has also been suggested to be involved in fatty acid-induced lipotoxicity^19,20^. Most trials increasing mitochondrial tricarboxylic acid (TCA) cycle metabolism can significantly protect against fatty acid-induced lipotoxicity while prohibiting TCA cycle metabolism induces toxicity similar to palmitate-induced lipotoxicity in beta cells^21,22^.

This study was initiated to investigate an assumption that chemical protecting palmitate-induced lipotoxicity could improve hyperglycemia in animal model of T2D through beta cell preservation. We found that sodium fluorocitrate (SFC), an inhibitor of TCA cycle enzyme aconitase, could strongly and specifically protect against palmitate-induced INS-1 beta cell death. To determine whether SFC could protect obesity-induced T2D, SFC (10 mg/kg) was injected to 8 weeks old db/db mice every other day for 10 weeks and plasma glucose and insulin levels were then measured. Beta cell preservation was also determined through investigating insulin- and cleaved caspase 3-stained pancreatic islets.

## Results

### Sodium fluorocitrate is protective against palmitate-induced INS-1 cell death

**Figure 1:**
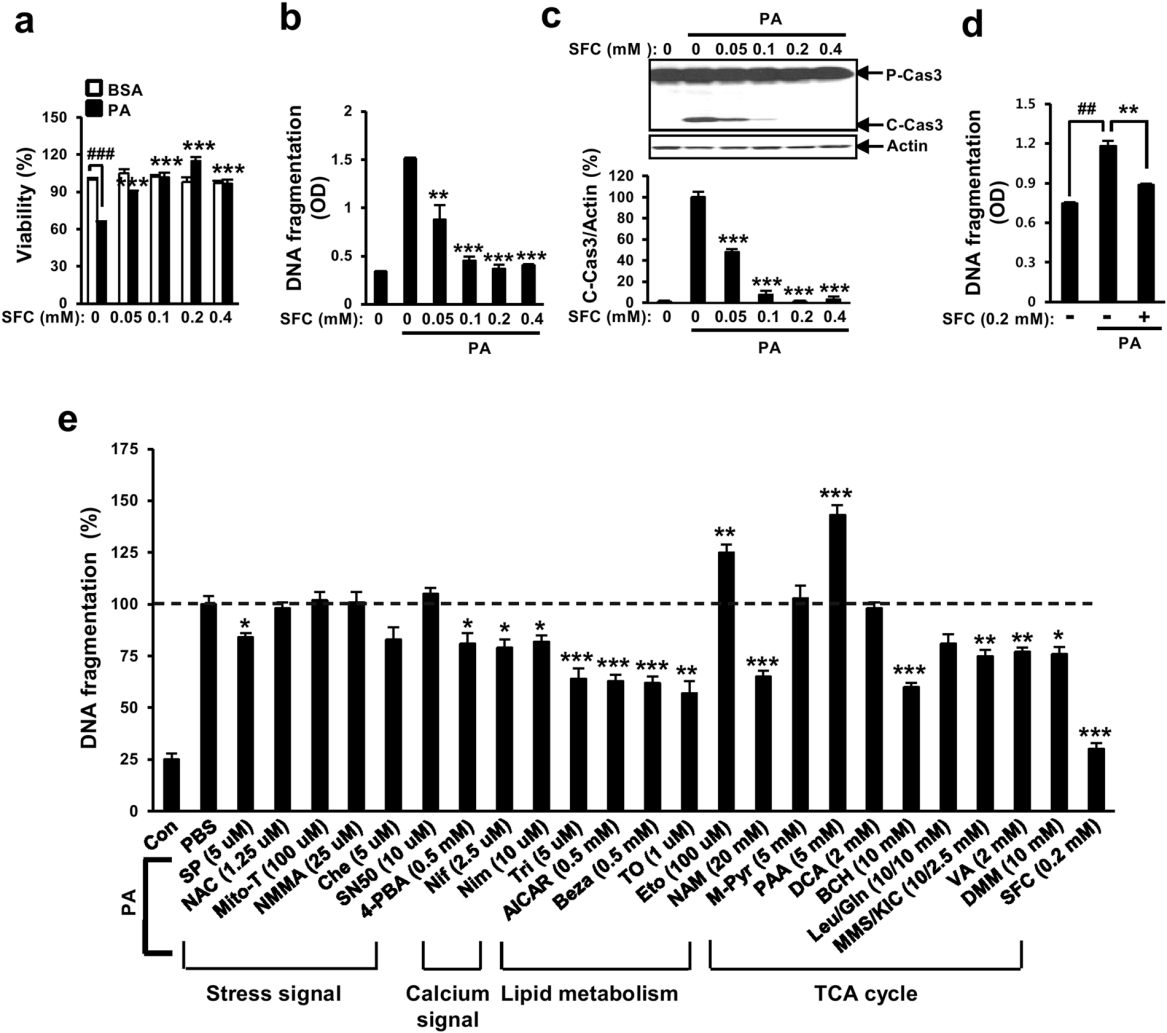
Sodium fluorocitrate protects against palmitate-induced death in INS-1 beta cells. INS-1 cells were treated with different concentrations of sodium fluorocitrate (SFC) in the presence of 0.4 mM palmitate (PA) for 16 h. (**a**) Viability was measured by MTT assay. (**b**) DNA fragmentation was measured with Cell Death Detection ELISA kit (Roche Applied Science, Mannheim, Germany). (**c**) Cleaved caspase 3 (C-Cas3) was measured by immunoblotting with anti-caspase 3 antibodies. Full-size original blots are presented in supplementary Fig. S6. (**d**) Islet cells isolated from db/db mice were treated with 0.2 mM SFC in the presence of 0.4 mM palmitate for 48 h. DNA fragmentation was measure with Cell Death Detection ELISA kit. Data are expressed as mean ± SE from three independent experiments and analyzed by one-way ANOVA (**b**,**c**,**d**) or two- way ANOVA (**a**). ^##^ *p* < 0.01; ^###^ *p* < 0.001 *vs*. BSA-treated cells. **p < 0.01; ***p < 0.001 *vs*. palmitate-treated cells. (**e**) INS-1 cells were treated with different signal/metabolism modulators in the presence of 0.4 mM palmitate for 16 h. DNA fragmentation was determined by using Cell Death Detection ELISA kit. Maximum DNA fragmentation obtained by palmitate treatment was designated as 100%. Relative reduction of DNA fragmentations by treatment with different modulators was described. Data are presented as mean ± SE and analyzed by one-way ANOVA. *p < 0.05; **p < 0.01; ***p < 0.01 vs. palmitate-treated INS-1 cells. Abbreviations for chemicals and their functions are included in supplementary abbreviations.

Mitochondrial metabolism was suspected to be involved in palmitate-induced lipotoxicity. Chemicals such as etomoxir and phenyl acetic acid (carnitine palmitoyltransferase-1 inhibitor and pyruvate carboxylase inhibitor, respectively) that could inhibit mitochondrial metabolism can augment palmitate-induced lipotoxcity. On the other hand, supplementation with TCA cycle intermediates (e.g., methyl pyruvate, leucine/glutamine, monomethyl succinate/a-ketoisocaproate, valeric acid, and dimethyl malate) for augmenting mitochondrial metabolism can protect against palmitate-induced lipotoxcity^21,22^. To further study the involvement of mitochondrial TCA cycle metabolism in palmitate-induced lipotoxicity, the effect of sodium fluorocitrate (SFC) known as aconitase inhibitor on palmitate-induced lipotoxicity was investigated. Unexpectedly, SFC demonstrated very strong protective effect on palmitate-induced beta cell lipotoxicity. SFC prevented viability reduction, DNA fragmentation, and caspase 3 cleavage in INS-1 beta cells induced by palmitate in a concentration-dependent manner (Fig. 1a,b and c). In particular, SFC at 0.2 mM showed the highest protective effect on palmitate-induced cell death. SFC at 0.2 mM restored palmitate-induced viability reduction, DNA fragmentation, and caspase 3 cleavage almost completely (Fig. 1a,b and c). SFC was also significantly protective against palmitate-induced death in primary islet cells isolated from db/db mice or Sprague Dawley rats (Fig. 1d and Supplementary Fig. S1). SFC itself used in this study did not seem to be toxic to INS-1 cells since SFC at 4 mM was not toxic (Supplementary Fig. S2a). On the other hand, SFC showed the strongest protective effect on palmitate-induced INS-1 cell death compared to other known anti-lipotoxic agents such as JNK inhibitor, ER chaperone, calcium blockers, AMPK activator, PPARα agonist, LXR agonist, nicotinamide, and glutamate dehydrogenase activator at their maximal protective concentrations (Fig. 1e). Treatment with 0.2 mM SFC restored palmitate-induced DNA fragmentation to palmitate-untreated normal level.

### Sodium fluorocitrate’s protective effect is specific for palmitate-induced lipotoxicity

**Figure 2:**
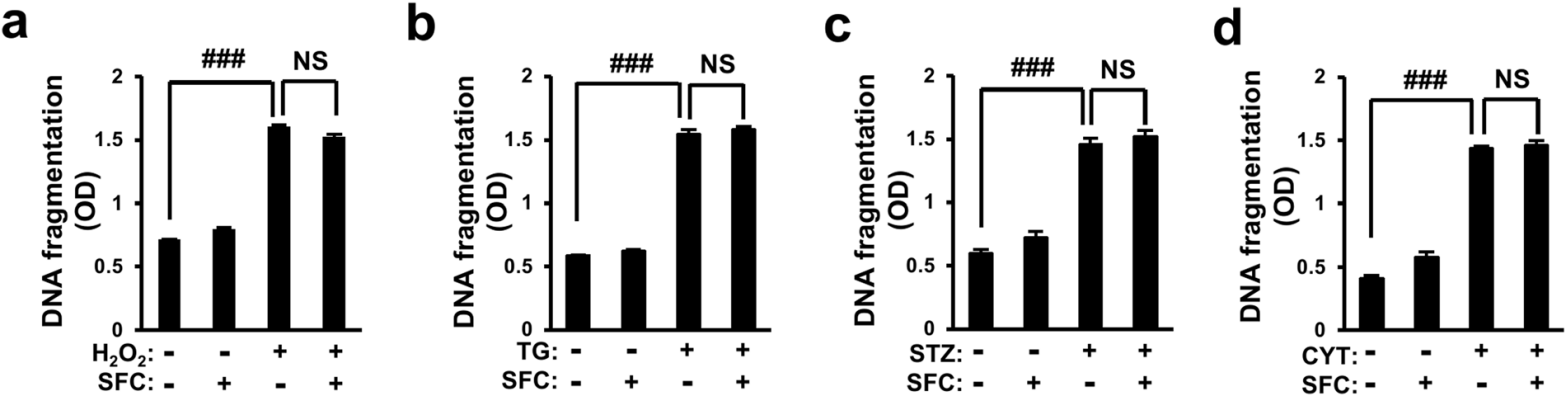
Protective effect of sodium fluorocitrate is specific for palmitate-induced lipotoxicity. INS-1 cells were treated with 0.2 mM sodium fluorocitrate (SFC) in the presence of (**a**) 50 μM H_2_O_2_ for 3 h, (**b**) 1 μM thapsigargin (TG) for 9 h, (**c**) 30 mM streptozotocin (STZ) for 12 h or (**d**) cytokine mixture (CYT; 5 ng/ml of IL-1β, 10 ng/ml of TNF-α, 50 ng/ml of INF-γ) for 24 h. Cell death was measured with Cell Death Detection ELISA kit. Data are presented as mean ± SE and analyzed by two-way ANOVA. ^###^ *p* < 0.001 *vs*. PBS-teated INS-1 cells. NS: not significant.

To determine whether SFC’s protective effect on palmitate-induced death was specific for palmitate-induced lipotoxicity, INS-1 cell death was induced by various beta cell-specific toxins and protective effects of SFC on cell death were then investigated. Oxidative and ER stress have been reported to play important roles in beta cell destruction in T2D^23^. Streptozotocin is known to be cytotoxic to beta cells and thus, it has been used for constructing an animal model of type 1 diabetes (T1D)^24^. Cytokine mixture has also been suggested as cytotoxic mediators for autoimmune destruction of beta cells in T1D^25^. In this study, INS-1 cell death was induced by treatment with hydrogen peroxide (H_2_O_2_) as a reactive oxygen species (ROS), thapsigargin as an inhibitor of sarco/endoplasmic reticulum calcium ATPase (SERCA), streptozotocin as a beta cell-specific toxin, or proinflammatory cytokine mixture (interleukin-1β, tumor necrosis factor-α, and interferon-γ) as beta cell specific-cytotoxic immune mediators. As shown in Fig. 2, all these beta cell-specific cytotoxic molecules increased DNA fragmentation in INS-1 cells. However, 0.2 mM SFC failed to protect DNA fragmentation increment in INS-1 cells induced by these agents (Fig. 2a,b,c and d). These data suggested that SFC’s protective effect on beta cell death was very specific for palmitate-induced lipotoxicity.

### Sodium fluorocitrate prevents palmitate-induced endoplasmic stress induction

**Figure 3:**
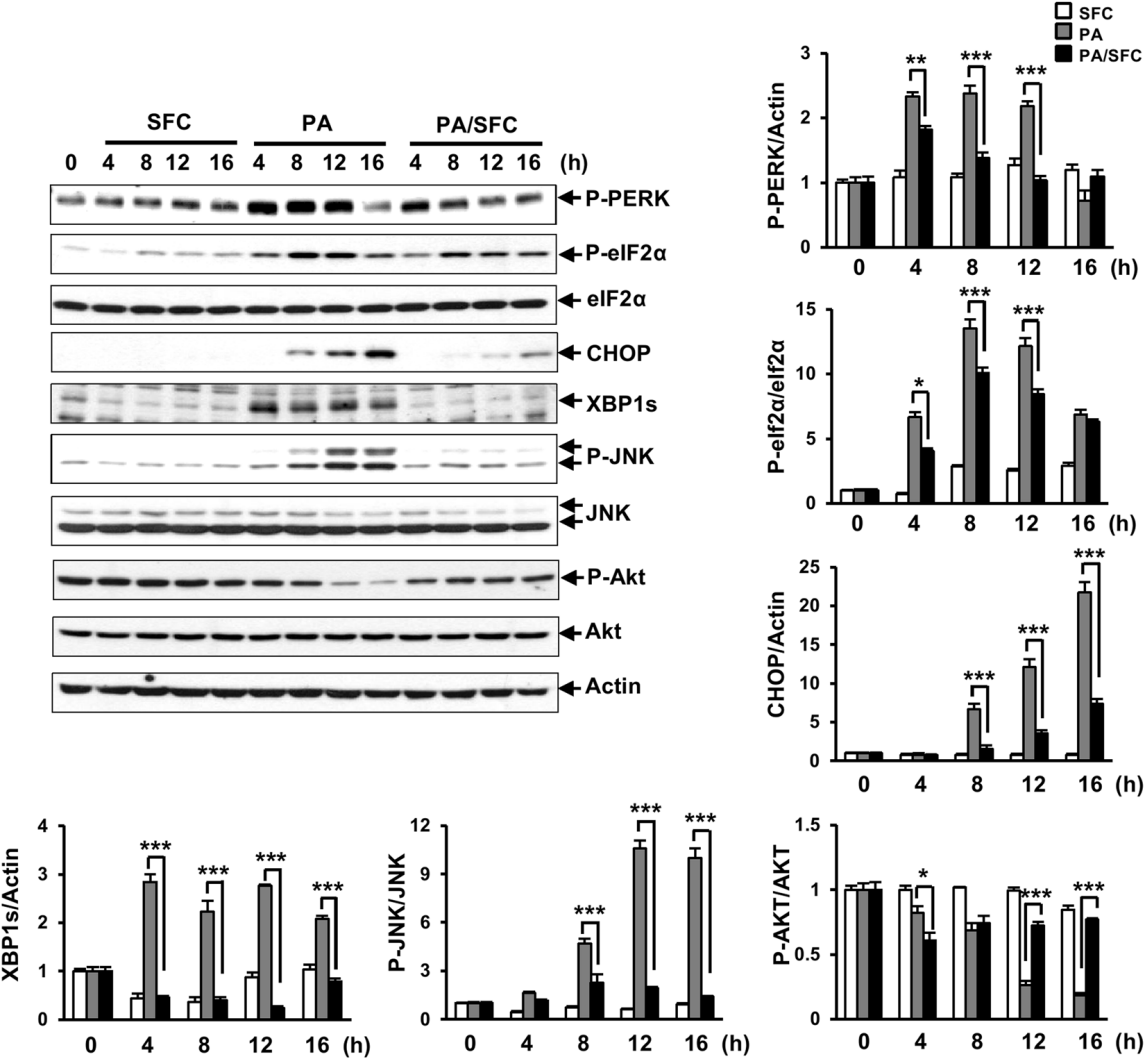
Effects of sodium fluorocitrate on palmitate-induced cellular signals. INS-1 cells were treated with 0.2 mM of sodium fluorocitrate (SFC) in the presence of 0.4 mM palmitate (PA) for the indicated time period. ER stress responses were analyzed by immunoblotting of phospho-protein kinase RNA-like ER kinase (P-PERK), phospho-eIF2α (P-eIF2α), C/EBP homologous protein (CHOP), spliced X-box binding protein 1 (XBP1s), and phospho-c-Jun N-terminal kinase (P-JNK) with anti-phospho-PERK, phospho-eIF2α, CHOP, XBP1, and phospho-JNK antibodies, respectively. Insulin signal was analyzed by immunoblotting of phospho-Akt (P-Akt) with anti-phospho-AKT antibody. Cropped blots are shown for clarity. Full-size original blots are presented in supplementary Fig. S6. Data are presented as mean ± SE and analyzed by two-way ANOVA. *p < 0.05; **p < 0.01; ***p < 0.001 *vs*. palmitate-treated INS-1 cells.

Stress response caused by accumulation of unfolded or mis-folded proteins in ER rumen has been reported to be a critical mediator in palmitate-induced lipotoxicity^13–15^. To determine whether SFC could prevent palmitate-induced ER stress responses in INS-1 beta cells, expression levels of ER stress markers such as phospho-PERK, phosph-eIF2a, CHOP, spliced XBP1, and phospho-JNK were investigated in palmitate-treated INS-1 cells with or without SFC treatment. As shown in Fig. 3, expression levels of phospho-PERK and phospho-eIF2a were increased at 4 h after treatment with 0.4 mM palmitate, peaking at 8 h after treatment. Levels of CHOP and phospho-JNK were increased at 8 h after palmitate treatment, peaking at 16 h and 12 h after treatment, respectively. Expression levels of spliced XBP were increased at all time points after palmitate treatment. SFC treatment significantly reduced expression levels of all ER stress markers induced by palmitate treatment at all indicated time points (Fig. 3). On the other hand, the level of phospho-Akt, a known survival signal in insulin signaling pathway, was significantly decreased at 12 h and 16 h after palmitate treatment. SFC also prevented palmitate-induced reduction of phospho-Akt levels at 12 h and 16 h after treatment (Fig. 3).

### The protective effect of sodium fluorocitrate on palmitate-induced INS-1 cell death is not due to its inhibitory effect on aconitase

**Figure 4:**
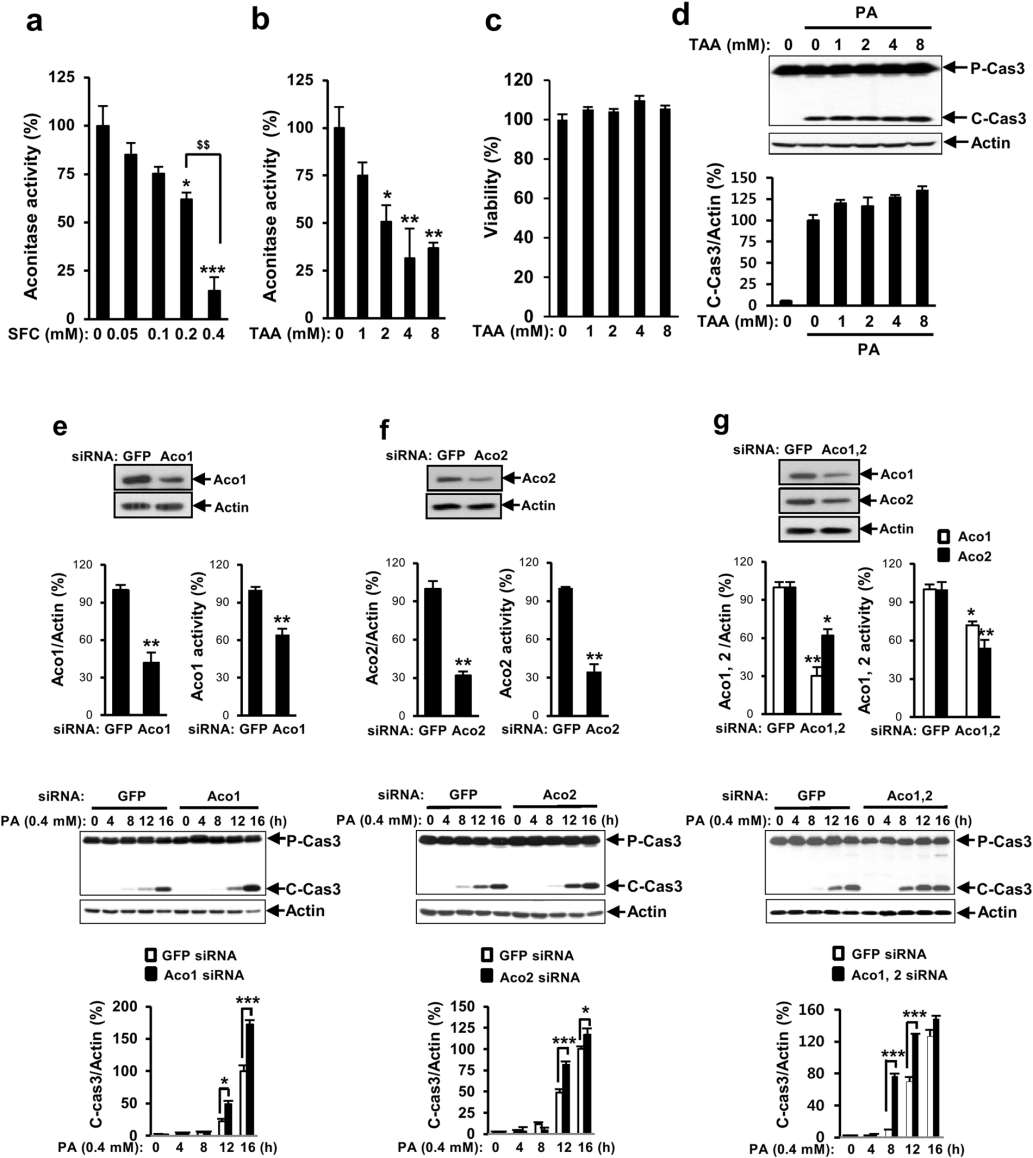
Aconitase inhibition does not protect palmitate-induced lipotoxicity. INS-1 cells were treated with different concentrations of sodium fluorocitarte (SFC) or trans-aconitic acid (TAA) for 8 h. (**a**,**b**) Aconitase activity was measured by aconitase activity assay kit. (**c**) Cell viability was measured by MTT assay. Data shown are relative activities. Data are expressed as mean ± SE and analyzed by one-way ANOVA. *p < 0.05; **p < 0.01; ***p < 0.001 *vs*. PBS-treated INS-1 cells. ^$$^ *p* < 0.01 *vs*. 0.2 mM SFC-treated INS-1 cells. (**d**) INS-1 cells were treated with 0.4 mM palmitate (PA) for 16 h in the presence of different concentrations of TAA. Cleaved caspase 3 levels were investigated by immunoblotting with anti-caspase 3 antibodies. Data are presented as mean ± SE and analyzed by one-way ANOVA. (**e**,**f**,**g**) INS-1 cells were transfected with siRNA of green fluorescent protein (GFP), aconitase 1 (Aco1), aconitase 2 (Aco2), or aconitase 1/aconitase 2 (Aco1, 2). After 36 h of incubation, knockdowns of aconitases were determined by immunoblotting with anti-Aco1 or Aco2 antibodies. Aconitase activities were measured by aconitase activity assay kit. Transfected INS-1 cells were treated with 0.4 mM palmitate for indicated time period. Cell death was determined by measuring levels of cleaved caspase 3 in immunoblotting with anti-caspase 3 antibodies. Full-size original blots are presented in supplementary Fig. S6. Data are presented as mean ± SE and analyzed by unpaired Student’s t-test (Immunoblotting for aconitases and activity for aconitases) or two-way ANOVA (Immunoblotting for cleaved caspase 3). *p < 0.05; **p < 0.01; ***p < 0.001 *vs*. siGFP-transfected INS-1 cells.

To determine whether inhibitory effect of aconitase was involved in SFC’s protective effect on palmitate-induced lipotoxicity, effects of another inhibitor or molecular down-regulation of aconitase on palmitate-induced lipotoxicity were investigated. Initially, inhibitory effect of SFC on aconitase was investigated using aconitase assay kit. As shown in Fig. 4a, SFC reduced aconitase activity in a dose-dependent manner. SFC at 0.2 mM, the concentration that showed the highest protective effect on palmitate-induced lipotoxicity (Fig. 1a), reduced aconitase activity by 38% compared to untreated control. SFC at 0.4 mM, the concentration that showed similar protective effect on palmitate-induced lipotoxicity compared to 0.2 mM (Fig. 1b and c), showed higher inhibitory effect on aconitatse activity than that at 0.2 mM. This result indicated that there was a discordance between concentrations showing protective activity of SFC against palmitate-induced lipotoxicity and its inhibitory activity against aconitase. On the other hand, treatment with trans-aconitic acid (TAA) as another aconitase inhibitor reduced the activity of aconitase in a dose-dependent manner without significantly decreasing cell viability (Fig. 4b and c). However, TAA at all concentrations failed to prevent palmitate-induced caspase 3 cleavage in INS-1 cells (Fig. 4d). Furthermore, knockdown of cytosolic aconitase (aconitase 1), mitochondrial aconitatse (aconitase 2), or double knockdown of aconitase 1 and aconitase 2 failed to protect palmitate-induced INS-1 cell death (Fig. 4e,f and g) in spite of their down-regulation of protein levels and enzymatic activities, indicating that the protective effect of SFC on palmitate-induced lipotoxicity was not due to its inhibitory effect on aconitase.

### Sodium fluorocitrate reduces cellular uptake of palmitate into INS-1 cells

**Figure 5:**
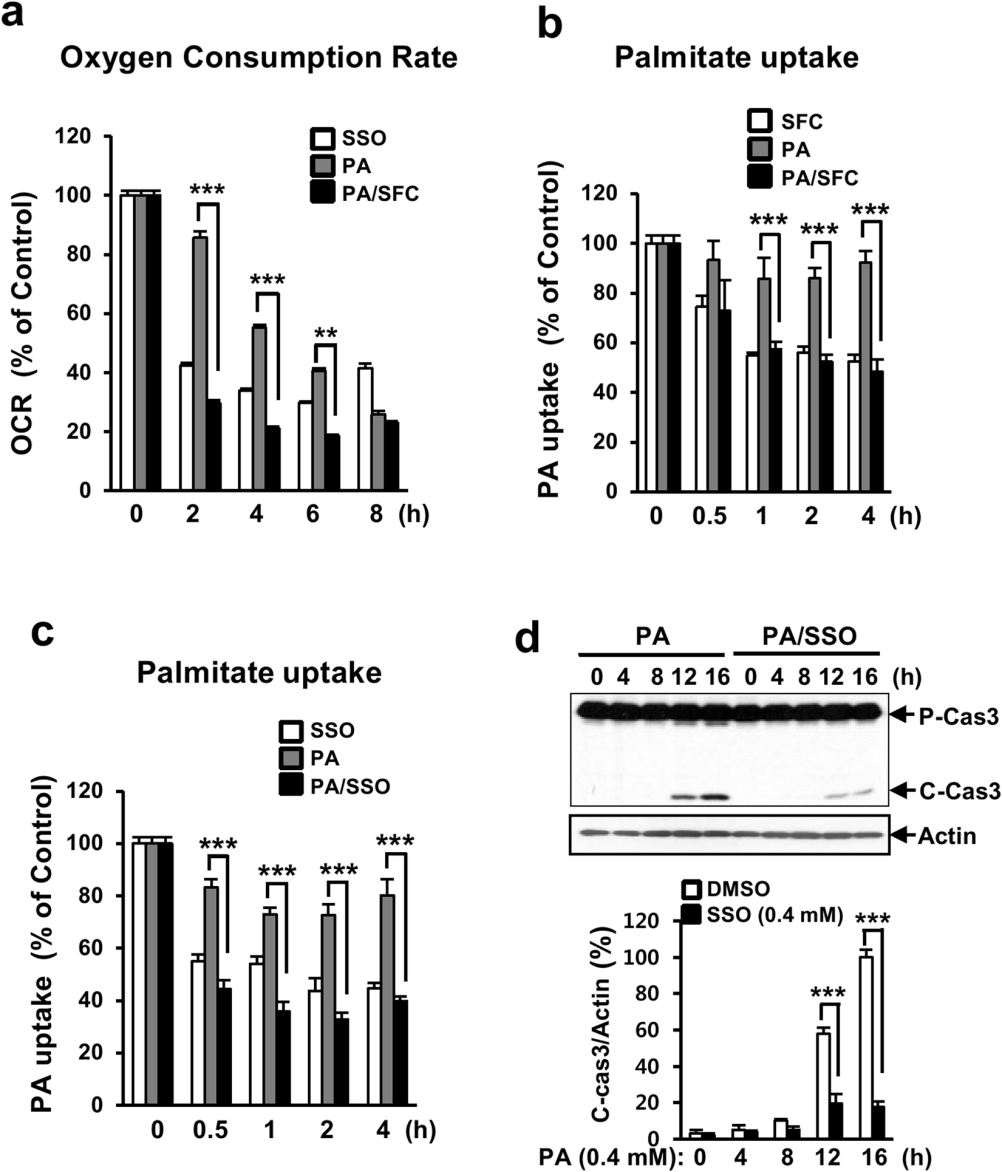
Sodium fluorocitrate reduces cellular uptake of palmitate in INS-1 cells. (**a**) INS-1 cells were treated with 0.2 mM sodium fluorocitrate (SFC) in the presence or absence of 0.4 mM palmitate (PA) for indicated time period. Oxygen consumption rate (OCR) of palmitate as a carbon substrate was determined by using Seahorse XF24 extracellular analyzer. Data are presented as mean ± SE from three independent experiments and analyzed by two-way ANOVA. **p < 0.01; ***p < 0.001 vs. palmitate-treated INS-1 cells. INS-1 cells were treated with 0.2 mM sodium fluorocitrate (SFC) (**b**) or 0.4 mM sulfo-N-succinimidyl oleate (SSO) (**c**) in the presence or absence of 0.4 mM palmitate (PA) for indicated time period. After treating with [^14^C]-palmitate for 30 min, radioactivities of [^14^C]-palmitate in cells were measured by using a beta counter. Data are expressed as mean ± SE and analyzed by two-way ANOVA. ***p < 0.001 *vs*. palmitate-treated INS-1 cells. (**d**) INS-1 cells were treated with 0.4 mM of palmitate in the presence of SSO for indicated time period. Levels of cleaved caspase 3 (C-Cas3) were analyzed by immunoblotting with anti-caspase 3 antibodies. Full size original blots are presented in supplementary Fig. S6. Data are presented as mean ± SE and analyzed by two-way ANOVA. ***p < 0.001 *vs*. DMSO-treated INS-1 cells.

Since the protective effect of SFC on palmitate-induced beta cell death was very specific for palmitate-induced lipotoxicity, we suspected that SFC’s protective effect on palmitate-induced lipotoxicity might be due to its inhibition on fatty acid metabolism. Fatty acid oxidation metabolism was initially investigated by measuring oxygen consumption rate (OCR) using palmitate as a carbon substrate. As shown in Fig. 5a, SFC significantly inhibited the oxidation of palmitate in INS-1 cells. In particular, SFC at 0.2 mM reduced the OCR by approximately 80% in INS-1 cells, indicating that SFC inhibited oxidation metabolism of palmitate and such inhibition was related to its protective effect on palmitate-induced lipotoxicity. Since the first step of fatty acid metabolism is cellular uptake of fatty acid into cytoplasm, we investigated whether SFC inhibited palmitate uptake in INS-1 cells. Palmitate uptake into INS-1 cells was determined by measuring radio activity of labeled palmitate existed in cytosolic fraction after treatment with ^14^C-palmitate. As shown in Fig. 5b, SFC significantly inhibited the uptake of labeled palmitate into INS-1 cells in the presence or absence of palmitate. SFC pre-treatment for 4 h reduced palmitate uptake by around 48% compared to untreated control. This result suggests that reduced fatty acid metabolism through reduced cellular uptake of palmitate plays a key role in the protective effect of SFC on palmitate-induced lipotoxicity. To confirm that reduced cellular uptake of palmitate was essential for the protective effect of SFC on palmitate-induced lipotoxicity, we examined the effect of sulfo-N-succinimidyl oleate (SSO) as another inhibitor of fatty acid uptake on palmitate-induced INS-1 cell death^26^. Treatment with 0.4 mM SSO inhibited the cellular uptake of palmitate into INS-1 cells and significantly prevented palmitate-induced caspase 3 cleavage (Fig. 5c and d).

### Sodium fluorocitrate improves hyperglycemia in db/db diabetic mice

**Figure 6:**
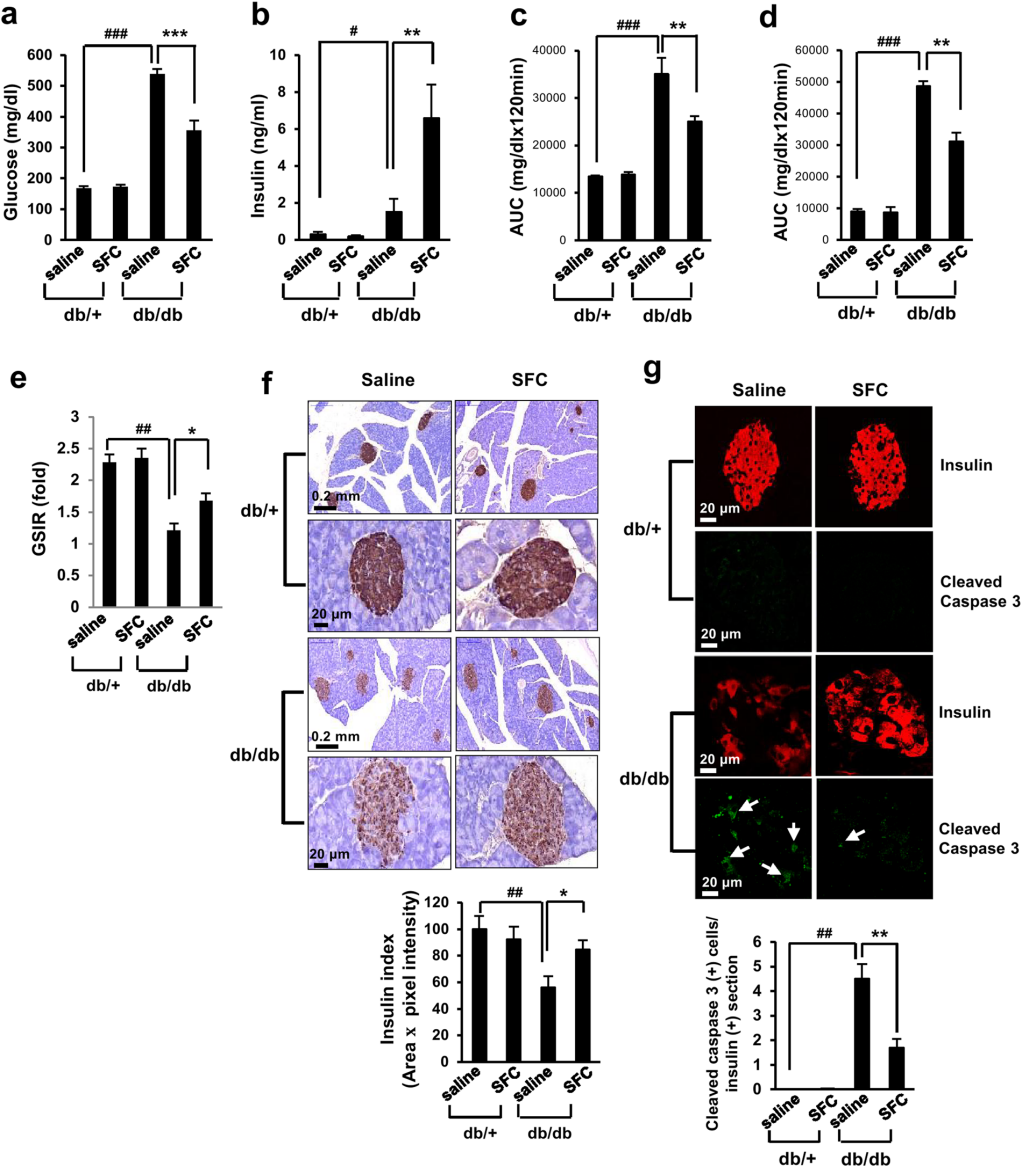
Sodium fluorocitrate improves hyperglycemia in db/db mice. Glucose levels (**a**) and insulin levels (**b**) in plasma after 6 h of fasting were determined in 18 weeks old db/db mice treated with SFC for 10 weeks (n = 10/group). (**c**,**d**) Area under the curves (AUCs) were obtained from intraperitoneal glucose tolerance test (GTT) and insulin tolerance test (ITT) (Supplementary Fig. S5a and S5b). (**e**) Glucose-stimulated insulin release (GSIR) was determined by fold-increase of insulin level at 30 min after glucose infusion (0.5 g/kg) in 14 h-fasted mice (n = 5/group). (**f**) Pancreatic sections were immuno-stained with insulin antibody/HRP/DAB system and counterstained with methylene blue. Insulin index was determined by multiplying stained area and pixel of intensity in insulin-positive islets (n = 10 islets/group). (**g**) Pancreatic sections were immuno-stained with a guinea pig anti-insulin antibody or rabbit anti-cleaved caspase 3 antibody. Secondary immune reactions were carried out with Alexa Fluor 594-conjugated goat anti-guinea pig IgG or Alexa Fluor 488-conjugated goat anti-rabbit IgG antibodies, respectively. Red 594 or green 488 fluorescences was visualized using confocal microscope. Beta cell apoptosis in islets was determined by measuring cleaved caspase 3-positive cells in insulin-stained area (n = 10 islets/group). Data are presented as mean ± SE and analyzed by one-way ANOVA. ^#^ *p* < 0.05; ^##^ *p* < 0.01; ^###^ *p* < 0.001 *vs*. saline-treated db/+ mice. **p < 0.01; ***p < 0.001 vs. saline-treated db/db mice.

In db/db diabetic mice, beta cell mass is gradually increased by 12 weeks of age. However, it starts to decrease after that^27^. Severe hyperglycemia in these mice is due to the loss of insulin-producing beta cells^27^. To determine whether SFC could preserve beta cells and improve hyperglycemia in these mice, SFC (10 mg/kg) was injected to 8 weeks old male db/db mice every other day for 10 weeks and plasma levels of insulin and glucose were then investigated. SFC used in these studies was not thought to be toxic because LD_50_ of SFC was close to 150 mg/kg (Supplementary Fig. S3a). In addition, mice treated with SFC for 10 weeks did not show any signs of intoxication such as loss of appetite, loss of weight, abnormality of skin and hair, abnormality of breath, or abnormality of motility. Interestingly, SFC treatment induced continuous weight gains in db/db mice, even at later stage (Supplementary Fig. S4a). Body weights of SFC-treated db/db mice were heavier than those of saline-treated db/db mice at 15, 16, 17, and 18 weeks of age. SFC treatment reduced water intake in db/db mice (Supplementary Fig. S4b). However, there was no significant difference in food intake between SFC-treated and saline-treated db/db mice (Supplementary Fig. S4c). As shown in Fig. 6a, plasma glucose level after 6 h-fasting was 538 mg/dL at 18 weeks of age. However, 10 weeks of treatment with SFC decreased the glucose level to 355 mg/dL at the same age. Improvement of hyperglycemia through SFC treatment may play a role in continuous increase of body weight and reduction of water intake without change of food intake in db/db mice. On the other hand, SFC treatment increased plasma insulin level to 6.6 ng/mL from 1.35 ng/mL at 18 weeks of age, suggesting that 10 weeks of treatment with SFC improved loss of insulin-secreting capability in db/db mouse beta cells (Fig. 6b). Glucose tolerance test (GTT) after 14 h-fasting demonstrated that SFC treatment significantly reduced glucose levels at 0.5, 1, and 2 h after glucose injection (Supplementary Fig. S5a). Insulin tolerance test (ITT) revealed that glucose levels at 0.5, 1, and 2 h in SFC-treated db/db mice were slightly lower than those in saline-treated db/db mice (Supplementary Fig. S5b). Area under the curves (AUCs) of both GTT and ITT were also significantly lower in SFC-treated db/db mice compared to saline-treated db/db (Fig. 6c and d). These data suggested that SFC improved both glucose tolerance and insulin tolerance. Since improvement of glucose tolerance and increase of plasma insulin in SFC-treated db/db mice strongly suggested prevention of beta cell failure, glucose-stimulated insulin releases (GSIRs) after SFC treatment was investigated in these mice. GSIR by glucose stimulation (0.5 g/kg) was significantly decreased in db/db mice compared to db/+ mice (Fig. 6e). However, SFC treatment increased GSIR by 37% compared to saline-treated db/db control (Fig. 6e). In accordance with improvement of GSIR, insulin index obtained by multiplication of islet area and intensity of insulin staining was significantly higher in SFC-treated db/db mice than that in saline-treated mice (Fig. 6f). Furthermore, architecture and contour of islets were more intact in SFC-treated mice (Fig. 6f). To determine whether SFC treatment could prevent apoptotic death in islet beta cells, the number of cells stained with anti-cleaved caspase 3 antibodies was counted in db/db mouse islets. The number of cells stained with both anti-insulin and anti-cleaved-caspase 3 antibodies was greatly increased in db/db mouse islets compared to db/+ islets. However, the number of cells stained with both antibodies was significantly lower in SFC-treated db/db islets than saline-treated db/db islets (Fig. 6g). Cells stained with both antibodies were rarely observed in db/+ mouse islets irrespective of SFC treatment. These data suggested that SFC treatment prevented beta cell loss in db/db mice.

## Discussion

In this study, SFC was found to be strongly and specifically protective against palmitate-induced INS-1 beta cell death. Although SFC is originally known as an inhibitor of aconitase, its inhibitory effect on aconitase is not involved in its protective effect on palmitate-induced lipotoxicity. On the other hand, its inhibitory effect on cellular uptake of fatty acid into beta cells was likely to be important for its protective effect. The anti-diabetic effect of SFC was investigated in db/db mice with severe hyperglycemia due to progressive loss of beta cells^28^. Our results showed that ten weeks of treatment with SFC reduced plasma glucose level and improved glucose tolerance in these mice. Furthermore, SFC prevented reduction of plasma insulin, GSIR, and islet insulin in db/db mice. These results suggested that SFC treatment was anti-diabetic in db/db mice mainly due to its preservative effect on beta cell loss.

Sodium fluoroacetate (SFA) was originally developed as a pesticide due to its characteristic of metabolic poison. It has been suggested that SFA’s toxic effect is caused by intracellular conversion to SFC through citrate synthase^29^. SFC is known to be an aconitase inhibitor that disrupts TCA cycle, accumulates citrate, inhibits phosphofructokinase in glycolysis, and ultimately induces energy loss^30^. Although SFA showed protective effect on palmitate-induced lipotoxicity in INS-1 cells, there was a concentration difference between SFC and SFA for optimal protection (Fig. 1 and Supplementary Fig. S2c). The optimal protective concentration of SFA was around twenty five times higher than that of SFC. Although SFA was less toxic to INS-1 beta cells than SFC (Supplementary Fig. S2a and S2b), SFA was more toxic to C57BL/6 J mouse (Supplementary Fig. S3a and S3b). The reason why SFC was less toxic to C57BL/6 J mouse than SFA was not determined in our study. Stronger toxic characteristic of SFA than SFC *in vivo* might be due to unknown toxic effect of SFA as well as inhibitory effect of SFC on aconitase. Different conversion rate of SFA to SFC between culture system and animal system or existence of different isomers in SFC might have contributed to differences in their toxicities.

There was discordance in SFC’s inhibitory effect on aconitase and its protective effect on palmitate-induced lipotoxicity according to its concentrations (Fig. 1b and Fig. 4a). TAA as another inhibitor of aconitase was never protective against palmitate-induced death. In particular, molecular knockdown of aconitases was not protective against palmitate-induced death either. These data suggest that SFC’s protective effect on palmitate-induced lipotoxicity was not due to its inhibitory effect on aconitase. On the other hand, metabolic inhibition of fatty acid might be involved in its protective effect on palmitate-induced lipotoxicity (Fig. 5a). Since the protective effect of SFC on palmitate-induced lipotoxicity was very specific and SFC inhibited most stress signals in palmitate-treated cells, it was suspected that SFC’s protective effect might be due to its inhibition at early stage of fatty acid metabolism. In fact, our experiments demonstrated that SFC inhibited cellular uptake of palmitate into INS-1 cells. Another fatty acid uptake inhibitor SSO had similar protective effect on palmitate-induced INS-1 cell death. All these results suggest that reduced metabolism of fatty acids through insufficient cellular uptake of palmitate might play a key role in SFC’s protective effect on palmitate-induced lipotoxicity. Since lipid accumulation through excessive uptake of fatty acids was a pathogenic cause of metabolic diseases such as non-alcoholic fatty liver disease, cardiomyopathy, and atherosclerosis^31–33^, inhibition of fatty acid uptake through SFC treatment might have therapeutic potential for preventing these diseases. On the other hand, it has been reported that SSO could not be used for therapeutic purpose of these metabolic diseases due to its chemical instability^34^.

Fatty acid-induced lipotoxicity has been thought to be due to activation of apoptotic signals through excessive activation of stress/inflammatory signals^35–37^. Most attempts to reduce stress/inflammatory signals (i.e., treatment with JNK inhibitor, calcium blockers, and chemical chaperones) significantly protected against palmitate-induced INS-1 cell death (Fig. 1e). Several modulators involved in metabolism of lipid such as AMPK activator, PPAR alpha agonist, LXR agonist, and glutamate dehydrogenase activator also protected against palmitate-induced lipotoxicity (Fig. 1e). Since the protective effects of the metabolic modulators on palmitate-induced lipotoxicty were generally stronger than those of stress signal inhibitors and the metabolic modulators could prevent palmitate-induced activation of stress signals^17,22,38^, palmitate-induced metabolic alteration might play a key role in palmitate-induced lipotoxicity through activating stress/inflammatory signals. Our data demonstrated that SFC fundamentally reduced cellular uptake of palmitate and prevented palmitate-induced activation of stress/inflammatory signals in INS-1 cells. In addition, it was shown that SFC’s protective effect on palmitate-induced lipotoxicity was stronger than any other anti-lipotoxic agents. These results are strongly in accord with the hypothesis that metabolism of fatty acids is involved in role in palmitate-induced lipotoxicity through stress/inflammatory signals. A recent report that chemical inhibition of fatty acid uptake can prevent palmitate-induced stress responses in beta cells also suggests involvement of fatty acid metabolism in activation of stress/inflammatory signals^39^.

Beta cell failure and insulin resistance are thought to be pathogenic causes of T2D. Current treatments for T2D are focused on improving insulin resistance and insulin secretion. In spite of these treatments, T2D patients need insulin treatment at later stage of the disease possibly due to loss of beta cells^40^. In this context, attempts to preserve beta cells would be a promising strategy to prevent severe progression of T2D^41–43^. Since fatty acid-induced lipotoxicity is thought to play a role in beta cell loss in T2D, chemicals that can protect fatty acid-induced lipotoxicity might be able to improve hyperglycemia through preserving beta cells in T2D. In fact, we found that SFC was highly and specifically protective against palmitate-induced death in INS-1 cells and it reduced hyperglycemia and improved glucose tolerance in db/db diabetic mice. SFC also greatly increased plasma insulin level with slight improvement of insulin resistance. These results suggested that maintenance of insulin secretion capacity even in insulin resistance played a role in improving hyperglycemia in severely diabetic subjects. Maintenance of islet integrity by SFC treatment also supports its role in beta cell preservation. Our results were similar to previous results showing that hyperglycemia was improved through beta cell preservation in GLP-1 analogue-treated db/db mice^44^. Taken together, our results supports the assumption that fatty acid-induced lipotoxicity in beta cells is involved in the progression of T2D.

In conclusion, our results revealed that SFC could inhibit cellular uptake of fatty acid into beta cells. It was strongly and specifically protective against palmitate-induced INS-1 beta cell death. SFC also improved hyperglycemia through preserving islet beta cells in db/db diabetic mice. SFC’s inhibitory characteristic of fatty acid uptake might be used to treat several metabolic disease in which excessive uptake of fatty acid might be involved, such as type 2 diabetes, non-alcoholic fatty liver disease, cardiomyopathy, and atherosclerosis.

## Methods

### Ethics statement

All experiments were performed in accordance with Ajou University Safety and Ethics guidelines. In particular, animal experiments were carried out according to the animal experiment procedure approved by Animal Ethics Committee of Ajou University (Permission number: 2013–0006).

### Reagents

Most chemicals used in this study were purchased from either Sigma–Aldrich (St. Louis, MO, USA) or Merck Bioscience (Darmstadt, Germany), including the followings: glucose, palmitate, 3-[4,5-dimethylthiazol-2-yl]-2,5-diphenyltetrazoilium bromide (MTT), DL-fluorocitric acid barium salt, 1,9-pyrazoloanthrone (SP600125), N-acetyl cysteine (NAC), ng-monomethyl-L-arginine (L-NMMA), chelerythrine, H2N-Ala-Ala-Val-Ala-Leu-Leu-Pro-Ala-Val-Leu-Leu-Ala-Leu-Leu-Ala-Pro-Val-Gln-Arg-Lys-Arg-Gln-Lys-Leu-Met-Pro-OH (SN50), 4-phenylbutyrate (4-PBA), 1,4-dihydro-2,6-dimethyl-4-(2-nitrophenyl)-3,5-pyridinedicarboxylic acid dimethyl ester (nifedipine), 1,4-dihydro-2,6-dimethyl-4-(3′-nitrophenyl)-3,5-pyridinedicarboxylic acid 2-methoxyethyl-1-methylethyl ester (nimodipine), triacsin C, 5-aminoimidazole-4-carboxamide-1-β-D-ribofuranoside (AICAR), bezafibrate, T0901317, etomoxir, nicotinamide, methyl pyruvate, phenyl acetic acid, dichloroacetic acid, 2-aminobicyclo[2.2.1]heptan-2-carboxylic acid (BCH), leucine, glutamine, monomethyl succinate, ketoisocaproate, valeric acid, dimethyl malate thapsigargin, N-(methylnitrosocarbamoyl)-α-D-glucosamine (streptozotocin), and hydrogen peroxide solution. Mito-TEMPOL was purchased from Alexis Biochemicals (San Diego, CA, USA). Cytokines such as IL-1b, TNF-a, and IFN-c were purchased from R&D Systems (Minneapolis, MN, USA). Sulfosuccinimidyl oleate sodium was purchased from Abcam (Cambridge, UK). These chemicals were dissolved in culture medium or dimethyl sulfoxide (DMSO). Anti-caspase 3, anti-phospho-PERK, anti-phospho-eIF2α, anti-eIF2α, anti-CHOP, anti-phospho-AKT, and anti-AKT antibodies were obtained from Cell Signaling Technology (Beverly, MA, USA). Anti-actin, anti-XBP1 and anti-JNK antibodies were purchased from Santa Cruz Biotechnology (Dallas, TX, USA). Anti-phospho-JNK antibody was obtained from Invitrogen (Carlsbad, CA, USA). Anti-aconitase 1 and anti-aconitase 2 antibodies were purchased from Abcam.

### Cells and culture

INS-1 rat insulinoma cells were maintained in RPMI 1640 medium supplemented with 10% FBS, 100 U/ml penicillin (Duchefa Biochemie, Haarlem, Netherlands), and 100 μg/ml streptomycin (Duchefa Biochemie) at 37 °C in a humidified atmosphere containing 5% CO_2_.

### Isolation of islets

Islets were isolated from 8 week-old male db/db mice using collagenase digestion method. Briefly, after injecting 3 ml of collagenase P (0.75 mg/ml) into bile ducts, each swollen pancreas was excised and incubated in a water bath at 37 °C for 7 min. After stopping collagenase digestion with cold Hanks’ balanced salt solution (HBSS), pancreatic tissues were disrupted by repetitive pipetting and subsequently passed through a 400-µm mesh. Islets were separated by centrifugation (1,700 g, 10 min) on 25%, 23%, 21.5%, and 11.5% Ficoll gradients. Islets at the interface between 21.5% and 11.5% fractions were collected and washed with HBSS. Healthy islets were hand-picked under a stereomicroscope. To obtain single beta cells, islets were treated with Trypsin-EDTA for 2 min and dissociated by repetitive pipetting. The dissociated islet cells were then cultured in RPMI 1640 medium containing FBS.

### DNA fragmentation assay

Cell death was determined by measuring fragmented DNAs using Cell Death Detection enzyme-linked immunosorbant assay (ELISA^plus^) kit (Roche Applied Science, Mannheim, Germany). Briefly, cells were lysed with lysis buffer supplied with the kit. After centrifugation (200 g, 10 min), the supernatant was pipetted onto an anti-streptavidin-coated microplate. Anti-DNA monoclonal antibody conjugated with peroxidase (anti-DNA-POD) and anti-histone-biotin was added. After incubation at 25 °C for 90 min, wells were rinsed with incubation buffer (supplied by the kit) three times. Color was developed by adding 2,20-azino-di-[3-ethylbenzthiazoline sulphonate] (ABTS) substrate solution followed by incubation with shaking at 250 rpm for 10–20 min. The amount of peroxidase retained in the nucleosome complex was determined by measuring the absorbance value at 405 nm on a microplate reader.

### Immunoblotting

Radioimmunoprecipition assay (RIPA) buffer containing 150 mmol/l NaCl, 1% NP-40, 0.5% deoxycholate, 0.1% sodium dodecyl sulfate, 50 mmol/l Tris.HCl at pH 7.5, and protease inhibitor cocktail (Roche Applied Science) was used to extract cellular proteins. Equivalent amounts of proteins (30 μg) in sodium dodecyl sulfate (SDS) sample buffer (50 mmol/l Tris-HCl at pH 6.8, 2% SDS, 100 mmol/l DL-dithiothreitol, 10% glycerol) were separated by 8–12% SDS-polyacrylamide gel electrophoresis (PAGE) and then transferred to polyvinylidene difluoride (PVDF) membrane (Millipore, Bedford, MA). Target antigens were reacted with primary antibodies after blocking PVDF membranes with 5% skimmed milk for 30 min. After binding with secondary antibodies (horseradish peroxidase-conjugated anti-mouse IgG or anti-rabbit IgG antibodies), immunoreactive bands were detected with enhanced chemiluminescence system (Pierce, Rockford, IL, USA). Band intensities were determined with densitometric analysis using one-dimensional Quantity One® 1D image analysis system (Bio-Rad, Hercules, CA).

### Aconitase assay

Aconitase activity was determined using aconitase activity colorimetric assay kit (Biovisoin, Milpitas, CA). Briefly, cells were scraped, washed with phosphate buffered saline (PBS), and homogenized in cold assay buffer supplied by the kit. Cytosolic fraction in supernatant after differential centrifugation (2000 g, 10 min) was collected and used to measure aconitase 1 activity. After centrifugation at 16,000 x g for 15 min at 4 °C, the pellet containing mitochondrial fraction was collected and dissolved in cold assay buffer followed by sonication for 20 sec. This solution was used to measure aconitase 2 activity. Activation solution supplied by the kit was added to the sample solution and incubated on ice for 1 h to activate aconitase. Activated samples were added into 96-well plate and sample reaction mix supplied by the kit was then added to each test samples. Enzymatic reaction was performed at 25 °C for 60 min. Color was developed by further incubation at 25 °C for 10 min after adding the developer. Absorbance was measured at 450 nm. The activity of aconitaste was determined by measuring the quantity of isocitrate based on isocitrate standard curve.

### Transfection of small interfering RNAs

Small interfering RNAs (siRNA) were designed and synthesized by Genolution Pharmaceuticals (Seoul, Korea) or Sigma-Aldrich. Their sequences were as follows: green fluorescent protein (GFP) (GenBank: GU983383), 5′-GUU CAG CGU GUC CGG CGA GTT; rat aconitase 1 (GenBank: NM_ 017321.1), 5′-GCA UGA AGG UUC AGA UAA AUU; rat aconitase 2 (Genbank: NM_024398.2), 5′-GAC UCA AGU GCA AGU CUC A[dT][dT]. INS-1 cells were transfected with siRNA duplex using Neon^TM^ electro-transfection system (Invitrogen). The siRNA duplex in R buffer supplied by the system was then transfected into 1 × 10^7^ INS-1 cells by electric shock at 1650 V for 10 ms. After the transfection, cells were seeded into each well of appropriate plate and cultured for 36 h.

### Palmitate uptake assay

Palmitate uptake was determined by measuring intracellular [1-^14^C] palmitate. Briefly, cells (3 × 10^5^) grown in plates were washed with KHB buffer (NaCl 111 mmol/l, KCl 4.7 mmol/l, MgSO_4_ 2 mmol/l, Na_2_HPO_4_ 1.2 mmol/l, glucose 2.5 mmol/l) and incubated with radio-labeled palmitate (0.1 μCi [1-^14^C] palmitate [50 mCi/mmol, PerkinElmar, Covina, CA, USA]) at 37 °C for 30 min. After washing with KHB, 1% triton × 100 was added to the culture and the mixture was incubated at 25 °C for 2 h. Lysed cells containing [1-^14^C] palmitate were transferred to a new tube and measured with a liquid scintillation counter (Tri-Carb 2100; Packard Instrument, Meriden, CT, USA). Radio-activities were normalized against intracellular protein content in cells.

### Animal studies

Five-week-old male C57BLKS/J-db/db and C57BLKS/J-db/+ mice were purchased from Japan SLC Inc (Shizuoka Ken, Japan). Mice were housed in a temperature-controlled room (22 ± 2 °C), with a light/dark cycle of 12 h/12 h and fed ad libitum. At 8 weeks of age, mice were randomly assigned into either saline control group or SFC-treated group. Treated groups were injected intra-peritoneally every other day with 10 mg/kg of SFC for 10 weeks. Blood was collected from tail. Glucose levels were measured using Accu-check (Korea Roche Diagnostics, Seoul, Korea). Plasma insulin levels were determined using insulin RIA kit (Linco Research, Billerica, MA, USA).

### Statistical analysis

All experiments were repeated at least three times. Data are expressed as mean ± SE. All data were analyzed with GraphPad Prism 6.0 (GrahPad software, San Diego, CA). Unpaired Student’s T-test was used for comparisons of two datasets. One-way or two-way analysis of variance (ANOVA) with Boferroni post hoc test was used for comparison of more than two datasets or groups. Statistical significance was accepted at *p* < 0.05.

Methods for preparation of palmitate and sodium fluorocitrate and measurement of cell viability, oxygen consumption rate, plasma insulin, glucose tolerance test, insulin tolerance test, and glucose-stimulated insulin release and method for immunostainings are included in Supplementary Methods.

## Electronic supplementary material

Sodium fluorocitrate having protective effect on palmitate-induced beta cell death improves hyperglycemia in diabetic db/db mice
