# Supplementary material for: Sodium fluorocitrate having protective effect on palmitate-induced beta cell death improves hyperglycemia in diabetic db/db mice
Source: Sci Rep. 2017 Oct 10;7:12916. doi: 10.1038/s41598-017-13365-5 (PMC5635019; doi:10.1038/s41598-017-13365-5)
Supplement: Supplementary file 1 — Sodium fluorocitrate having protective effect on palmitate-induced beta cell death improves hyperglycemia in diabetic db/db mice [file 41598_2017_13365_MOESM1_ESM.pdf]

Supplementary Information

Sodium fluorocitrate having protective effect on palmitate-induced beta cell death improves hyperglycemia in diabetic db/db mice

Ik-Rak Jung, Sung-E Choi, Seung A Hong, Yoonjung Hwang, Yup Kang

Supplementary Methods

Preparation of palmitate. Palmitate/bovine serum albumin (BSA) conjugates were prepared by soaping palmitate with sodium hydroxide and mixing with BSA. Palmitate (20 mmol/l in 0.01 mol/l NaOH) was incubated at 70 ^{o}C for 30 min. These fatty acid soaps were then complexed with 5% BSA in phosphate-buffered saline (PBS) in a 1:3 volume ratio. Complexed fatty acids consisted of palmitate (5 mmol/l) and BSA (3.75%). These palmitate/BSA conjugates were diluted in 10% fetal bovine serum (FBS, Sigma-Aldrich)-containing culture medium and administered to cultured cells.

Preparation of sodium fluorocitrate. Sodium fluorocitrate was prepared from barium fluorocitrate (Sigma-Aldrich). Briefly, barium fluorocitrate (100 mg) was dissolved in deionized water (1.2 ml) by addition of 60.58 µl HCl (37%). Anhydrous sodium sulfate (51.12 mg) and anhydrous sodium carbonate (38.16 mg) were sequentially added to the fluorocitrate solution. Insoluble barium sulfate was removed by centrifugation. Sodium fluorocitrate solution was prepared at a final concentration of 0.1 M at pH = 7 ± 1.

Viability assay. Briefly, cells were seeded into 96-well cell culture plates and treated with MTT (0.5 mg/ml) at 37 °C for 2 hrs. Supernatants were discarded and 100 µl of acidic isopropanol (0.04 mol/l HCl) was then added to each well. After incubating at room temperature for 30 min, absorbance was measured at wavelength of 570 nm using a microplate reader (BIO-RAD, Hercules, CA, USA).

Oxygen consumption rate (OCR). INS-1 cells were seeded into XF24 cell culture microplates at a density of 5×10^4 cells/well with RPMI 1640 culture medium and allowed to attach overnight. Prior to XF assay, cells were prewashed three times with XF assay medium supplemented with glucose (2.5 mmol/l)/ carnitine (50 mmol/l)/ palmitate (0.2 mmol/l) for palmitate OCR and then equilibrated in XF assay medium at 37°C in a CO_2-free incubator for 1 h. Average oxygen consumption rate was obtained from three measurements.

Insulin measurement. Plasma insulin was measured using Merck Millipore rat insulin RIA kit (Billerica, MA, USA). Briefly, blood obtained from mouse tail was immediately centrifuged at 3,000g for 10 min at 4 ^{o}C. Upper plasma was collected and stored at -80 ^{o}C. Plasma was mixed with ^{125}I-insulin and anti-insulin antibody followed by incubation at 4 ^{o}C overnight. Precipitating reagents supplied from kit were incorporated to these mixtures. Reactants were further incubated at 4°C for 20 min. Aggregates were collected by centrifugation at 2,000 g for 20 min. Radio-activities in pellets were measured using a gamma counter (Perkin-Elmer, Fermont, CA, USA). The amount of insulin was then calculated using a standard curve.

Glucose tolerance and insulin tolerance test. To assess glucose tolerance, mice were first subjected to 14 h of fasting and injected intraperitoneally with glucose (0.5 g/kg of body weight). Blood was collected from tail at 0, 30, 60, and 120 min after glucose infusion. To assess insulin sensitivity, mice were subjected to 6 h of fasting and injected intraperitoneally with insulin (2 U/kg of body weight). Blood was collected from tail at 0, 30, 60, and 120 min after insulin infusion. Glucose levels (mg/dl) in blood were then measured by using Accu-

chek (Korea Roche Diagnostics, Seoul, Korea).

Glucose-stimulated insulin release. Glucose-stimulated insulin release was measured in SFC-treated and untreated db/db mice. Briefly, glucose (0.5 g/kg) was intraperitoneally injected into db/db mice after 14 h of fasting. Blood was collected from tail at 0 and 30 min after glucose infusion. Plasma was then collected by centrifugation (3,000 g, 10 min). Insulin levels (mg/dl) in plasma were then measured using Merck Millipore rat insulin RIA kit.

Immunostaining of insulin and cleaved caspase 3. . At 18 weeks of age, the pancreas of mouse was fixed in 10% neutral-buffered formalin and embedded in a paraffin block. These blocks were serially sectioned at a thickness of 5 μm using a rotary microtome. Sections mounted on glass slide were deparaffinized and blocked with 10% goat serum in PBS for 1 h. For insulin staining, a guinea pig anti-insulin antibody (DAKO, Carpinteria, CA, USA) diluted 1:500 in blocking buffer was overlaid on these sections for 3 h. After incubation with biotinylated goat anti-guinea pig IgG antibody, sections were reacted with streptavidin-conjugated horse radish peroxidase (DAKO). Immunoreactions were visualized by adding peroxidase substrate solution containing diamino benzidine tetra hydrochloride (DAB). After counter-staining with methylene blue, images were taken with an Olympus B201 microscope under bright-field illumination. Beta cell integrity in islet was determined by measuring insulin index. Insulin index was obtained by multiplying the area of insulin-stained islets and the pixel of intensity. For fluorescent staining of cleaved caspase 3 or insulin, sections were overlaid with a guinea pig anti-insulin antibody (DAKO) or rabbit anti-cleaved caspase 3 antibody (Cell Signaling Technology), respectively. Secondary immune reactions were carried out with Alexa Fluor 594-conjugated goat anti-guinea pig IgG (Thermo Fisher

Scientific, Waltham, MA, USA) or Alexa Fluor 488-conjugated goat anti-rabbit IgG (Thermo Fisher Scientific), respectively. Red 594 (excitation at 590 nm and emission at 617 nm) or green 488 (excitation at 495 nm and emission at 519 nm) fluorescence were visualized using confocal microscope (Zeiss 710).

Supplementary Abbreviations

PBS: phosphate buffered saline; SP: SP600125 as a JNK inhibitor; NAC: N-acetyl cysteine as an antioxidant; Mito-T: Mito-TEMPO as a mitochondria-targeted antioxidant; NMMA: L-NG-monomethyl-L-arginine as an iNOS inhibitor; Che: chelerythrine as a pan-PKC inhibitor; SN50 as a NFκB inhibitor; 4-PBA: 4-phenylbutyrate as a chemical chaperone; Nif: nifedipine as a voltage-dependent calcium channel blocker; Nim: nimodipine as a calcium channel blocker; Tri: triacin C as a acyl-CoA synthetase inhibitor; AICAR: 5-aminoimidazole-4-carboxamide ribonucleoside as a AMPK activator; Beza: bezafibrate as a PPARα agonist; TO: T0901317 as an LXR agonist; Eto: etomoxir as a CPT-1 inhibitor; NAM: nicotinamide; M-pyr: methyl-pyruvate; PAA: phenyl acetic acid as a pyruvate carboxylase inhibitor; DCA: dichloroacetic acid as a pyruvate dehydrogenase kinase inhibitor; BCH: 2-aminobicyclo (2.2.1) heptane 2-carboxylic acid as a glutamate dehydrogenase activator; Leu/Gln: leucine/glutamine as TCA cycle intermediate supplement; MMS/KIC: monomethyl succinate/a-ketoisocaproate as TCA cycle intermediate supplement; VA: valeric acid as a supplement of succinic acid; DMM: dimethyl malate as a malate supplement. SFC: sodium fluorocitrate as an aconitase inhibitor.

Supplementary Figures

Supplementary Figure S1

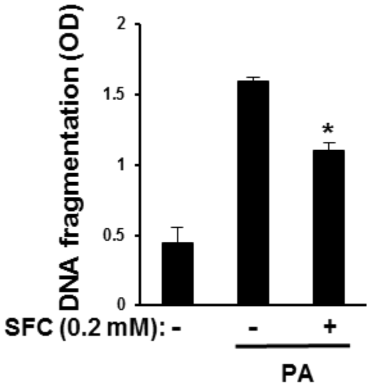

Islet cells isolated from Sprague Dawley rats were treated with 0.2 mM SFC in the presence of 0.4 mM palmitate (PA) for 60 h. DNA fragmentation was measure with Cell Death Detection ELISA kit. Data are expressed as mean ± SE from three independent experiments and analyzed by one-way ANOVA. *$p < 0.05$ *vs.* palmitate-treated rat islet cells.

Supplementary Figure S2

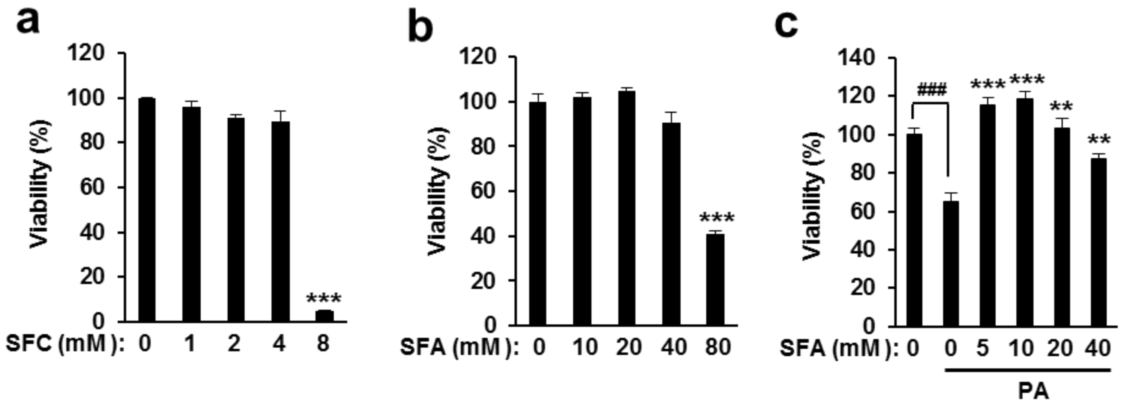

INS-1 cells were treated with different concentrations of sodium fluorocitrate (SFC) (**a**) or sodium fluoroacetate (SFA) (**b**) in the presence or absence of 0.4 mM palmitate (PA) (**c**) for 16 h. Cell viability was determined using MTT viability assay. Data are expressed as mean ± SE from three independent experiments and analyzed by one-way ANOVA. $^{###}p < 0.001$ *vs.* PBS-treated INS-1 cells. $^{**}p < 0.01$; $^{***}p < 0.001$ *vs.* palmitate-treated INS-1 cells.

Supplementary Figure S3

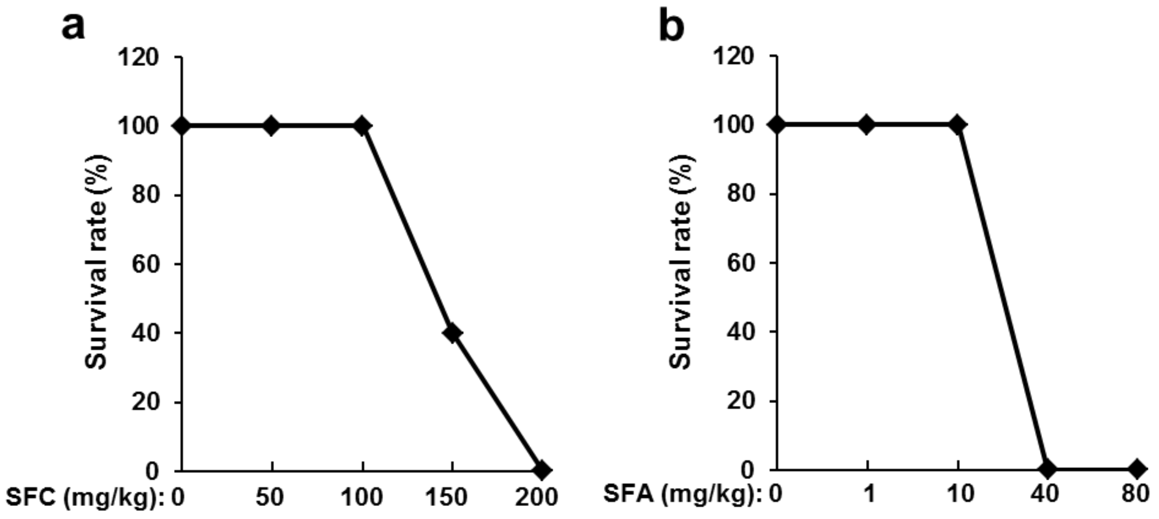

Different doses of sodium fluorocitrate (SFC) (**a**) or sodium fluoroacetate (SFA) (**b**) were intraperitoneally injected into C57BL/6J mice (n = 5). Survival of mice was determined at one day after injection. Single injection of 200 mg/kg of SFC resulted in 0% survival rate while single injection of 150 mg/kg of SFC resulted in 40% survival rate. Single injection of 100 mg/kg of SFC did not influence survival rate.

Supplementary Figure S4

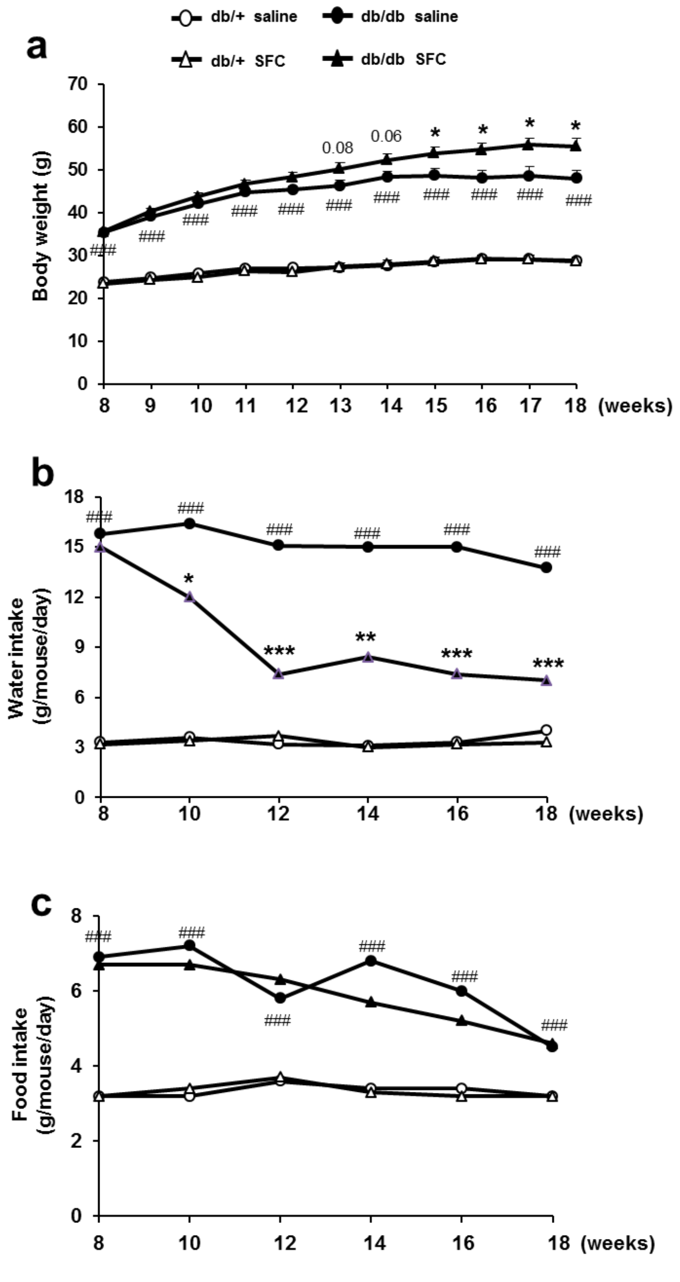

SFC (10 mg/kg) was injected into 8 week-old db/+ normal (n = 5) or db/db diabetic mice (n = 5) every other day for 10 weeks. Body weights (**a**) were measured every week. Water intake (**b**) and food intake (**c**) were measured every other week. Data were analyzed by one-way ANOVA. $^{###}p < 0.001$ *vs.* saline-treated db/+ mice. $^{*}p < 0.05$; $^{**}p < 0.01$; $^{***}p < 0.001$ *vs.* SFC-treated db/db mice.

Supplementary Figure S5

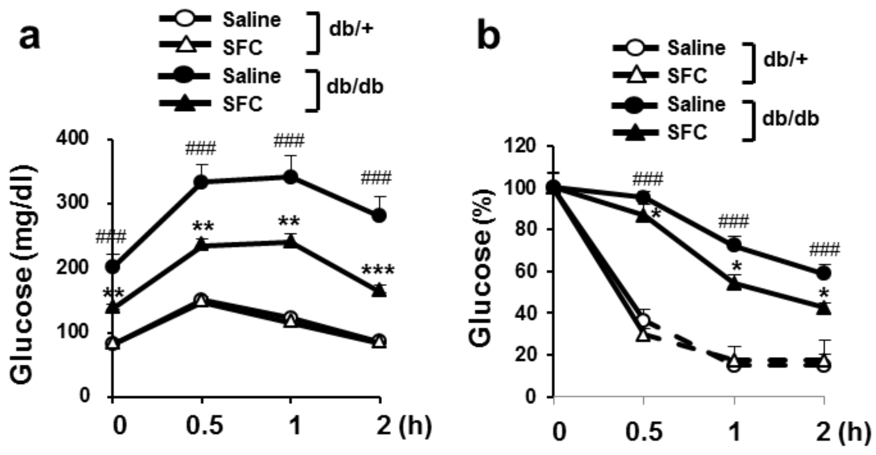

(**a**) Glucose tolerance test (GTT) was performed on overnight-fasted (14 h) mice by injecting glucose (0.5 g/kg body weight) (n = 10). (**d**) Insulin tolerance test (ITT) was performed on acute-fasted (6 h) mice by injecting insulin (2 U/kg body weight) (n = 10). Glucose concentrations were measured using Accu-check at indicated times. Data are presented as mean ± SE and analyzed by one-way ANOVA. $^{###}p < 0.001$ *vs*. saline-treated db/+ mice. $*p <$ 0.05; $**p < 0.01$ vs. saline-treated db/db mice

Supplementary Figure S6

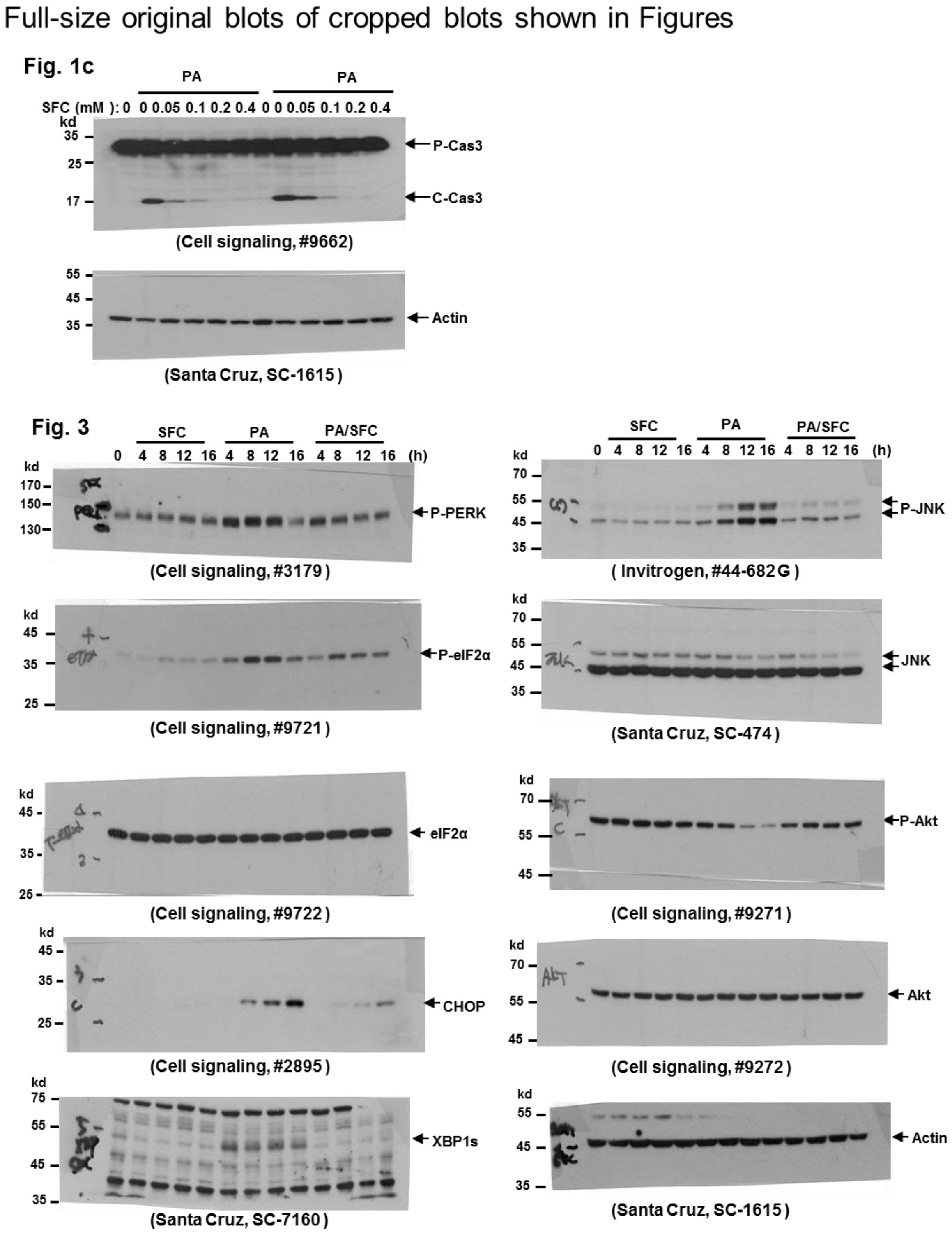

Full-length blots for cropped blots in Fig. 1c and Fig. 3.

Supplementary Figure S6 continued

Fig. 4d

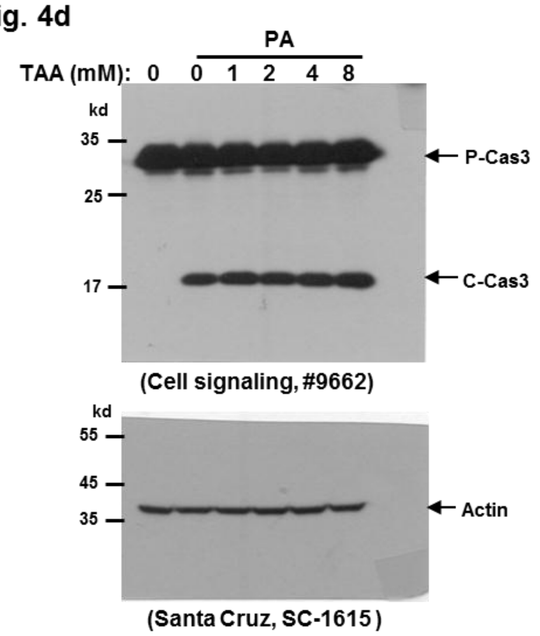

Fig. 4e

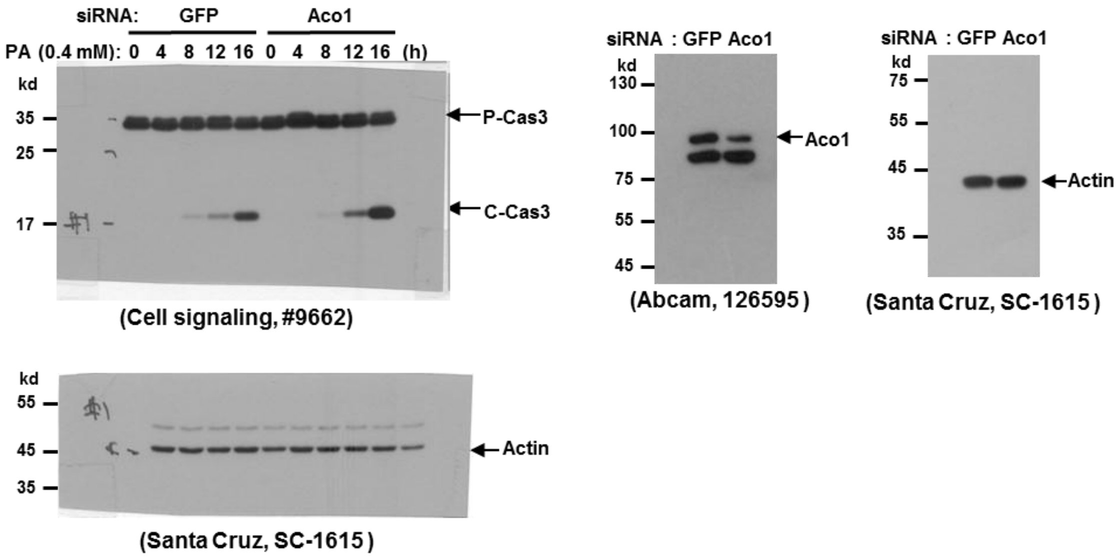

Full-length blots for cropped blots in Fig. 4d and 4e.

Supplementary Figure S6 continued

Fig. 4f

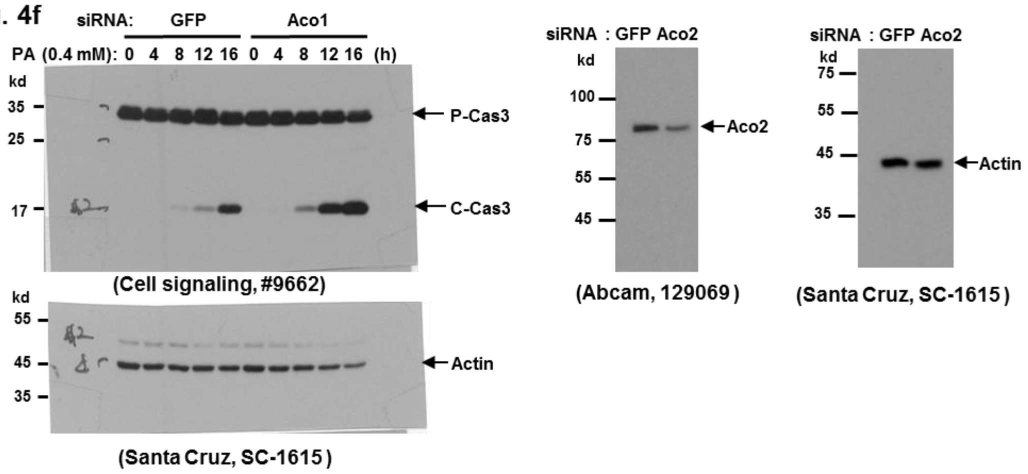

Fig. 4g

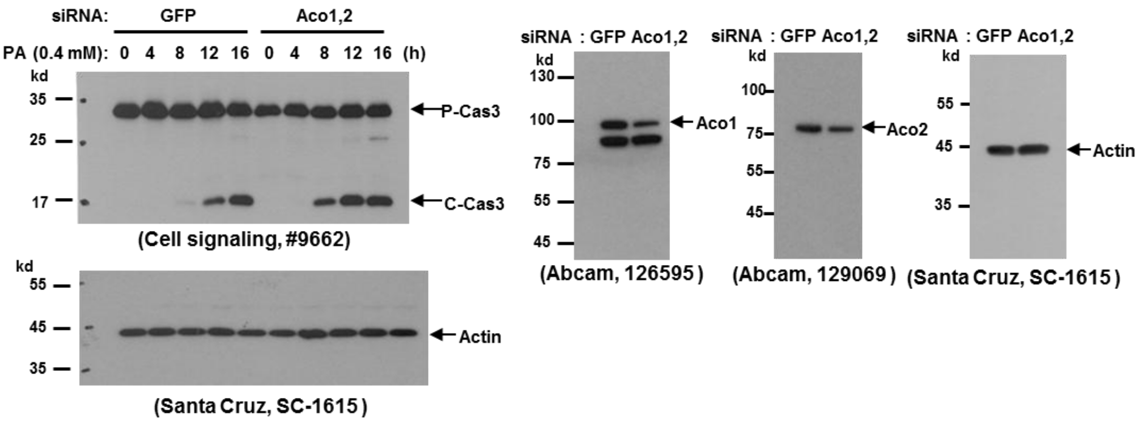

Fig. 5d

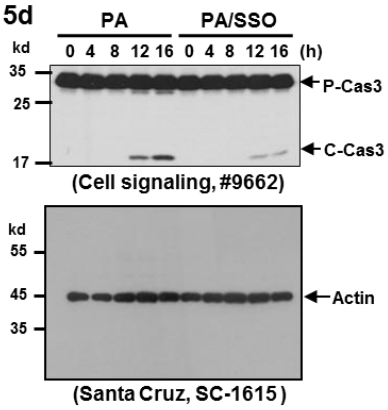

Full-length blots for cropped blots in Fig. 4f and 4g and Fig. 5d.
